# The effect of music intervention in decreasing pain and anxiety during outpatient hysteroscopy procedure: a systematic review and meta-analysis of randomized control trials

**DOI:** 10.1186/s12905-023-02489-8

**Published:** 2023-07-05

**Authors:** Mohamed Abd-ElGawad, Nada K. Abdelsattar, Mohamed Abdelmonem Kamel, Youstina Amin Sabri, Ethar Mohamed Fathy, Noha Abd El-Moez, Yasmeen Saeed Abdellatif, Ahmed A Metwally

**Affiliations:** 1 Faculty of Medicine, Fayoum University, Fayoum, Egypt; 2 Faculty of Pharmacy, Fayoum University, Fayoum, Egypt; 3 Department of Obstetrics and Gynecology, Faculty of Medicine, Cairo University, Cairo, Egypt

**Keywords:** Music, Hysteroscopy, Anxiety, And pain

## Abstract

**Background:**

Hysteroscopy is a common outpatient procedure but procedural pain limits its use. Music could be used as a pain-relieving intervention. We performed a systematic review and meta-analysis to investigate the effect of music on pain and anxiety during outpatient hysteroscopy.

**Methods:**

Four electronic databases were searched: PubMed, Scopus, Web of Science, and Cochrane Library, from inception to September 2022. We included only the Randomized Controlled Trials (RCTs) that investigated the effect of music on women who underwent outpatient hysteroscopy in reducing pain and anxiety levels compared to no music. We assessed the quality of included RCTs using the risk of bias tool 1 reported in the Cochrane Handbook of Systematic Reviews of Interventions. Data were pooled as the Mean Differences (MDs) with a 95% Confidence Interval (CI) in a random-effects model, using Review Manager 5.3 software. Also, we assessed the evidence of the results using the Grading of Recommendations Assessment, Development and Evaluation (GRADE).

**Results:**

Three RCTs (540 women) were included. Music significantly reduced visual analogue scale (VAS) pain scores as well as State-Trait Anxiety Inventory (STAI) scores compared to controls (MD = -1.28; 95% CI [-2.19, -0.36]; P = 0.007) and (MD = -3.91; 95% CI [-6.98, -0.85]; P = 0.01) respectively. Also, the decrease in VAS score for pain was significantly greater in the music group (MD = 1.44; 95% CI [0.44, 2.45]; P = 0.005). However, the change in STAI showed no significant difference between the two groups. The GRADE ratings for all outcomes were very low.

**Conclusion:**

Music is a potentially promising method for controlling pain for patients undergoing outpatient hysteroscopy; however, its effect in controlling anxiety is controversial.

## Introduction

Endoscopy ignited a revolution in different gynecological procedures [1]. It markedly replaced invasive surgical interferences with a minimally invasive outpatient procedure [2].

Hysteroscopy is a diagnostic tool used for several gynecological examinations that requires exploration of the uterine cavity, in addition to treatment of some localized pathologies without the need for extensive surgeries [3]. Based on the Ontario Ministry of Health and Long-Term Care claims database, over 10 000 Diagnostic Hysteroscopies are performed in Ontario annually [4]. Nguyen et al. reported that hysteroscopy is the most frequently performed procedure as an alternative to hysterectomy for Benign Gynecologic Conditions, with a utilization rate of 27.9% [5]. Economic analysis of hysteroscopy utilization revealed that the global hysteroscopy procedures market size is expected to reach USD 6.34 billion by 2028, registering a compound annual growth rate (CAGR) of 6.7% over the forecast period [6].

Hysteroscopy is considered a gold standard diagnostic and therapeutic procedure in different pathologies associated with the uterine cavity and cervical canal abnormalities both in premenopausal and postmenopausal women [7, 8]. Examples of These pathologies are uterine bleeding, endometrial polyps, submucous myomas and fertility status [7, 8]. A recent RCT investigated the safety of outpatient hysteroscopy in patients with failed in vitro fertilization and they did not report any hysteroscopy-related complications which clarified the safety of hysteroscopy use [9]. Hysteroscopy is used as an outpatient/office procedure with efficacy similar to using hysteroscopy under anesthesia [10].

Despite that hysteroscopy is simple, non-invasive, and does not need analgesia which makes it a safe outpatient procedure; pain and patients’ compliance are still obstacles to its usage [11]. Pain is the major complaint by women during outpatient hysteroscopy and it is the main reason for failure to complete the procedure [12]. The contributed factors of pain during the procedure include nulliparous women, previous cesarean delivery, menopausal status, chronic pelvic pain, and anxiety [13]. A review reported that outpatient hysteroscopy was associated with a higher level of anxiety compared to hysteroscopy done under general anesthesia [14]. A systematic review and meta-analysis studied the effect of pharmacological therapy for pain relief during hysteroscopy but it did not find a significant effect of pharmacological anesthesia during the procedure. Music could be used as a non-pharmacological analgesic by helping the patient to relax and be less stressed [15]. The World Health Organization recommended the use of music to control labor pain [16]. Generally, the reason behind the analgesic effect of music could be through distracting attention from the painful stimulus and focusing on the relaxing state of music [17]. Brain magnetic resonance imaging during applying a painful stimulus resulted in observing differences in the neural activity of different areas in the brain and brain stem [18]. Also, music was associated with dopamine release in the striatum which also can decrease pain perception [19].

Many Randomized Clinical Trials (RCTs) investigated the effect of music on relieving pain in different gynecological procedures; however, the results were controversial in these studies [20–22]. Mak et al. reported that music had no significant effect on patients’ perceived level of pain for patients undergoing outpatient hysteroscopy and colposcopy [22]. On the other hand, Law et al. and Angioli et al. concluded that music significantly lowered the level of pain perceived by patients during outpatient hysteroscopy [20, 21].

In view of the above-mentioned dispute, we present a systematic review and a meta-analysis of all the relevant literature and summarize the current evidence-based knowledge on the potential effect of music as a pain and anxiety reliever for patients undergoing outpatient hysteroscopy. If music showed a significant effect on lowering pain, it will increase compliance and satisfaction of patients with outpatient hysteroscopy. Also, it may abolish the need to use anesthesia and avoid its side effects. Therefore, The main objectives of this study are to determine if music can affect anxiety levels and perception of pain in women undergoing outpatient hysteroscopy.

## Methods

We reported a systematic review and meta-analysis according to the updated Preferred Reporting Items for Systematic Reviews and Meta-Analyses (PRISMA) statement [23]. As our study was a systematic review, ethical approval was not required and the registration of the study protocol was not obligate. Therefore, we did not register an online protocol for our study.

### Literature search strategy

We performed a comprehensive search of four electronic databases: PubMed, Scopus, Web of Science, and Cochrane Central Register of Controlled Trials (CENTRAL) from inception to September 2022. Our Population of interest were women who underwent outpatient hysteroscopy, the intervention was music in comparison with no music group, outcomes were pain and anxiety, and the included studies were RCTs. We used a combination of the following keywords to build the search strategy (Hysteroscopy OR OPH OR “Outpatient hysteroscopy” OR Uteroscopy OR Uterine endoscopy) AND (pain OR Anxiety) AND (music OR Symphony OR Rhythm OR Orchestra OR Song).

### Inclusion and exclusion criteria

We included only RCTs that investigated the effect of music or songs on women undergoing outpatient hysteroscopy in reducing pain or anxiety levels compared to no music. The exclusion criteria were: (1) non-randomized studies; (2) thesis and conference proceedings; (3) reviews, (4) non-English studies, and (5) observational studies. The primary outcomes were the endpoint measurement of the Visual Analogue Scale (VAS) for pain and the change of it from baseline. The secondary outcomes were the endpoint State-Trait Anxiety Inventory (STAI) score for anxiety and the change of it from the baseline measurement.

### Study selection and data extraction

Four authors independently screened the titles and abstracts of retrieved records according to the inclusion criteria then the included studies underwent full-text screening to check eligibility. Any debates were resolved by discussion.

Also, four authors independently extracted the data from included studies. The extracted data included body mass index (BMI), age, operative time, education, and types of procedures. Moreover, we extracted outcome measures such as the STAI score for anxiety assessment and the VAS score for pain. Finally, the quality assessment of included studies was performed by two authors and another author conducted the analysis.

### Quality assessment

We assessed the quality of included RCTs according to the Cochrane Handbook of Systematic Reviews of Interventions using Risk of Bias tool 1 which admits the following six domains: random sequence generation, allocation concealment, blinding of participants and personnel, blinding of outcome assessment, incomplete outcome data, selective outcome reporting, and other potential sources of bias. Authors judged domains and categorized them as “Low risk”, “High risk” or “Unclear risk of bias” [24]. Also, the evidence of the outcomes was independently assessed using the Grading of Recommendations Assessment, Development and Evaluation (GRADE) to know the probability of our results being far from the actual results [25].

### Publication bias

According to Egger et al., publication bias assessment was not reliable for less than ten pooled studies. Therefore, in the present study, we could not assess the existence of publication bias by Egger’s test or by funnel plot asymmetry [26].

### Data synthesis

We used Review Manager 5.3 for Windows in data analysis. Continuous data were pooled as Mean Difference (MD) with a 95% Confidence Interval (CI) in a random effect model as we observed heterogeneity in our outcomes. The heterogeneity was measured by the Cochrane Q test and I-square statistic and the results were considered to be significantly heterogeneous when the P value < 0.1 and I^2^ ≥ 50% [27]. The levels of heterogeneity were determined according to the Cochrane Handbook by low if I^2^ = 25%, moderate if I^2^ = 50%, and high if I^2^ = 75% [27]. We could not perform sensitivity analysis to exclude the most responsible article for increasing the heterogeneity level as the analysis included a limited number of studies. The results were considered to be significant if the P value was < 0.05.

## Results

**Fig. 1:**
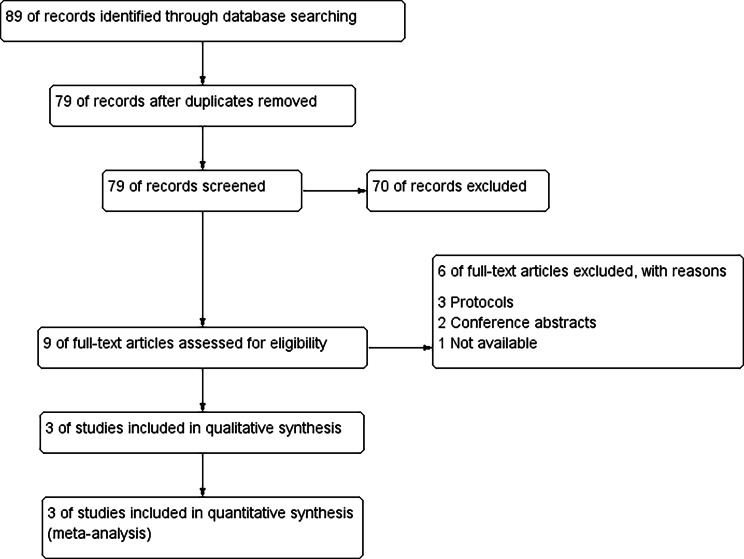
PRISMA flow diagram

The total records were 89 records after the initial database search (Pubmed revealed 16 records, Scopus revealed 33, web of Science revealed 18 and Cochrane revealed 22 records). Of these, ten duplicated records were removed using Endnote software. Upon screening of the title and abstract of the remaining 79 records, nine records were eligible. After reading their full texts, we excluded six ineligible studies according to inclusion criteria. The study flow diagram of the search results and study selection is illustrated in Fig. 1.

Finally, three RCTs with 540 women were included (268 in the music group and 272 in the control group) [20–22]. The study country, mean age, BMI, operative time, education level, mean decrease in VAS score for pain, decrease in STAI and the types of procedures are summarized in Table 1.

**Table 1:** General and baseline characteristics

Study ID	Study Design	Country	Study groups	Sample size	Age (mean ± SD)	BMI (mean ± SD)	Operative time (mean ± SD)	Education	Types of procedure	Decrease in Visual analogue scale score for pain	Decrease in state anxiety score (STAI)	Conclusion
Angioli et al. 2013	RCT	Italy	Music	176	57.03 ± 12.24	26.36 ± 4.52	15.95 ± 3.48	53 primary, 21 secondary, 69 High school, and 33 University	63 diagnostic and 37 operative	3.27 ± 3.81	7.09 ± 14.59	Music reduce anxiety and pain
			Standard	180	55.11 ± 13.91	26.28 ± 3.42	16.94 ± 5.05	47 primary, 41 secondary, 65 High school, and 27 University	67 diagnostic and 33 operative	1.11 ± 2.97	11.57 ± 6.79	
Mak et al. 2017	RCT	Netherlands	Music	39	45.4 ± 13.2	27.6 ± 7.6	NR	NR	23 diagnostic, 23 biopsies, and 45 therapeutic	2.79 ± 3.65	6.3 ± 12.8	Music does not reduce anxiety and pain
			Standard	42	45.2 ± 15	26.7 ± 6.4	NR	NR	31 diagnostic, 21 biopsies, and 48 therapeutic	1.94 ± 3.88	5.7 ± 10.9	
Law et al. 2021	RCT	China	Music	53	50.19 ± 11.74	25.86 ± 5.64	8.02 ± 6.36	11 primary, 31 secondary, and 11 tertiary	45 diagnostic and 8 operative	1.05 ± 3.13	NR	Music reduce pain
			Standard	50	47.88 ± 11.04	25.27 ± 4.17	8.86 ± 5.34	2 no education, 11 primary, 23 secondary, and 14 tertiary	44 diagnostic and 6 operative	0.23 ± 3.33	NR	

SD = Standard Deviation, BMI = Body Mass Index, and RCT = Randomized Clinical Trial

Table (1) shows the general and baseline characteristics of the included studies

### Risk of bias assessment

**Fig. 2:**
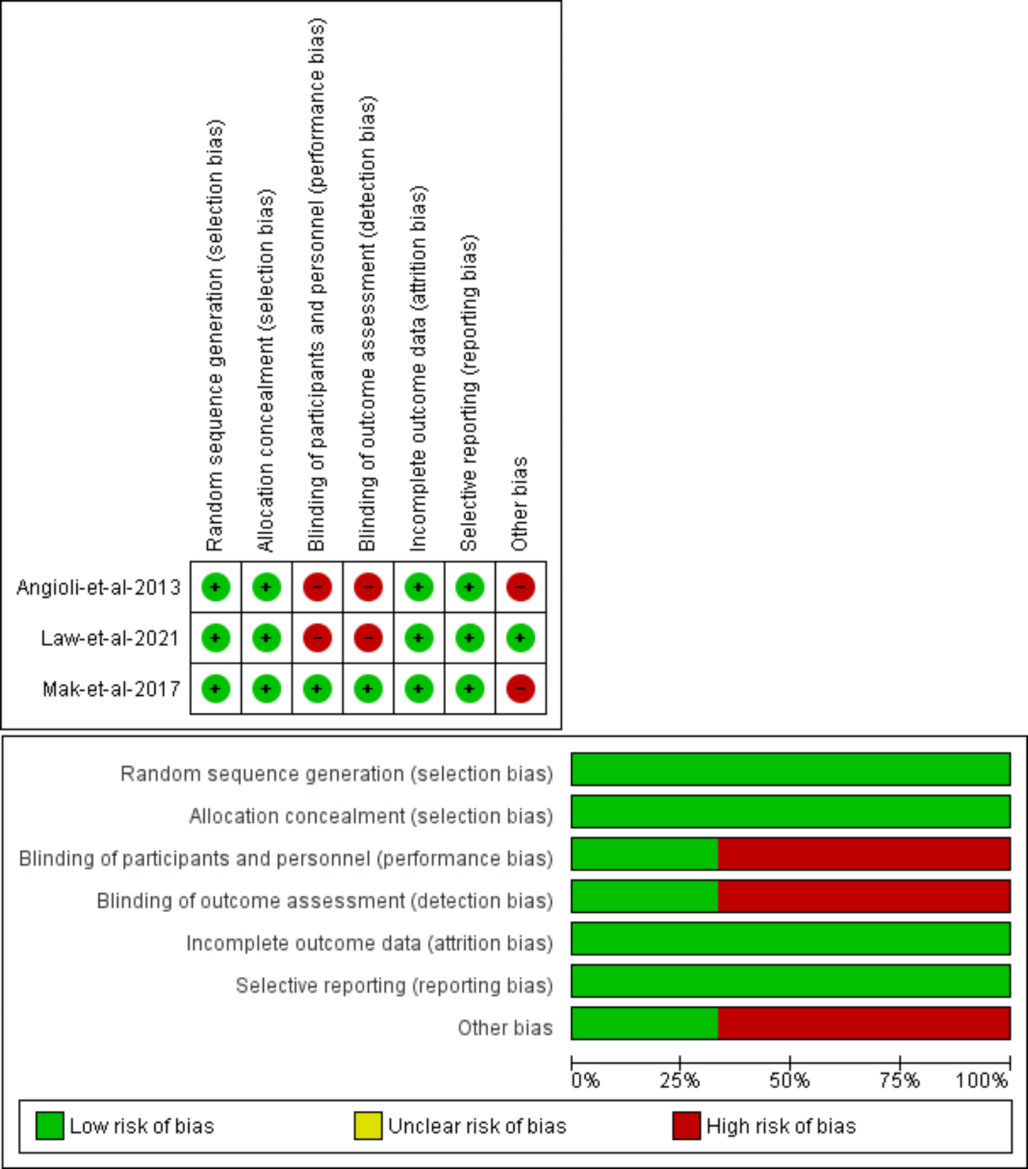
, Risk of bias graph and summary

The risk of bias graph and summary are summarized visually in Fig. 2.

#### Selection bias

All included studies had efficient randomization methods [20–22]. In addition, we reported good allocation concealment by using sealed numbered opaque envelopes in all included studies [20–22].

#### Performance and detection Bias

Law et al. 2021 and Angeioli et al. 2013 were considered at high risk of performance and detection bias due to the inability to blind neither the participants nor the outcome assessors because of the nature of the intervention [20, 21]. However, Mak et al. 2017. had a low risk of bias because the investigators did not tell the participants the nature of the intervention they were investigating [22].

#### Attrition bias

We considered all studies of low risk of bias as no missing data were reported in all studies [20–22].

#### Reporting bias

All studies reported the results of the outcomes that they were intending to measure in their protocols [20–22]. For studies that did not have an online protocol, the methods section in their paper was used and compared against the result Sects [20–22].

#### Other bias

Angeioli et al. 2013 had a high risk of bias because we could not find the protocol from the mentioned protocol code [20]. In Mak et al. 2017., there was a difference in experience between the doctors of both groups also waiting time and duration time were not measured [22].

### Outcomes

#### Endpoint VAS score for pain

**Fig. 3:**
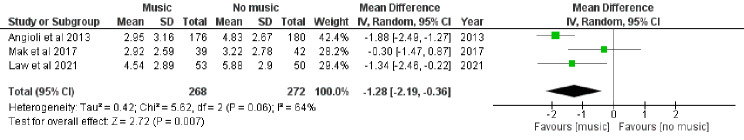
Endpoint Visual analogue scale score for pain

Three studies with 540 patients (268 in the music group and 272 in the control group) reported endpoint VAS scores for pain [20–22]. Music was significantly associated with lower VAS pain scores compared to the control group (MD = -1.28; 95% CI [-2.19, -0.36]; P = 0.007) [20–22]. The combined studies showed moderate heterogeneity (I² = 64%, P = 0.06), as shown in Fig. 3.

### Decrease in VAS score for pain from baseline

**Fig. 4:**
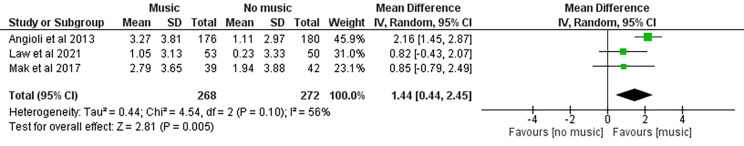
Decrease in Visual analogue scale score for pain

Three studies reported the decrease in VAS score for pain from baseline with 540 patients (268 in the music group and 272 in the control group) [20–22]. The decrease in VAS from baseline in the music group was significantly greater compared to the non-music group (MD = 1.44; 95% CI [0.44, 2.45]; P = 0.005) [20–22]. The combined studies showed moderate heterogeneity (I² = 56%, P = 0.10), as shown in Fig. 4.

### Endpoint STAI score for anxiety

**Fig. 5:**
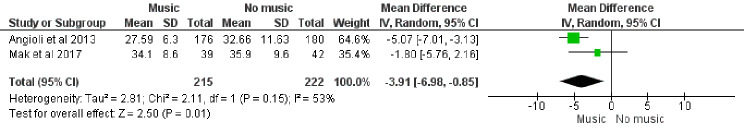
Endpoint State-Trait Anxiety Inventory (STAI)

Two studies with 437 patients (215 in the music group and 222 in the control group) reported the endpoint STAI scores for anxiety [20, 22]. Music was significantly associated with lower endpoint STAI scores compared to the control group (MD = -3.91; 95% CI [-6.98, -0.85]; P = 0.01) [20, 22]. The combined studies showed moderate heterogeneity (I² = 53%, P = 0.15), as shown in Fig. 5.

### Decrease in STAI score for anxiety

**Fig. 6:**
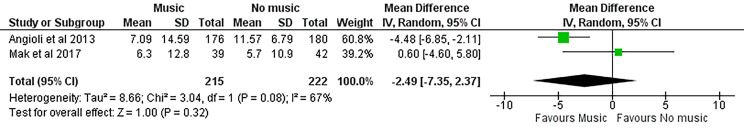
Decrease in state anxiety score (STAI)

Two studies with 437 patients (215 in the music group and 222 in the control group) reported the decrease in STAI scores for anxiety [20, 22]. The pooled analysis showed no significant difference between the two groups (MD = -2.49; 95% CI [-7.35, 2.37]; P = 0.32) [20, 22]. The combined studies showed moderate heterogeneity (I² = 67%, P = 0.08), as shown in Fig. 6.

### Summary of the finding with the GRADE assessment of the reported outcomes (Table 2)

We evaluated the evidence of all reported outcomes by the GRADE criteria. We found that all the outcomes had very low evidence which meant we had very low confidence about these results and the actual results had a high chance to be different from ours. These low evidence scores were due to the higher risk of bias, imprecision, and the presence of significant heterogeneity.

**Table 2:** Summary table and GRADE rating

Outcome name	Number of included studies	Study design	MD, 95% CI	Heterogeneity	Patients in the Music group	Patients in control	Risk of bias	Imprecision	heterogeneity	Indirectness	Other considerations^a^	Quality Judgement
Endpoint VAS score for pain	Three studies with 540 patients	RCTs	-1.28, (-2.19, -0.36)	I^2^ = 64%, P = 0.06	268	272	Serious^b^	Serious^c^	Serious^d^	Not serious	Not present	Very low ⊕◯◯◯
Decrease in VAS score for pain from baseline	Three studies with 540 patients	RCTs	1.44, (0.44, 2.45)	I^2^ = 56%, P = 0.10	268	272	Serious^b^	Serious^c^	Serious^d^	Not serious	Not present	Very low ⊕◯◯◯
Endpoint STAI score for anxiety	Two studies with 437 patients	RCTs	-3.91, (-6.98, -0.85)	I^2^ = 53%, P = 0.15	215	222	Serious^b^	Serious^c^	Serious^d^	Not serious	Not present	Very low ⊕◯◯◯
Decrease in STAI score for anxiety	Two studies with 437 patients	RCTs	-2.49, (-7.35, 2.37)	I^2^ = 67%, P = 0.08	215	222	Serious^b^	Serious^c^	Serious^d^	Not serious	Not present	Very low ⊕◯◯◯

RCTs = Randomized Control Trials, VAS = Visual Analogue Scale, STAI = State-Trait Anxiety Inventory, CI = Confidence Interval., MD = Mean Difference.

^a^ Other considerations are publication bias, large effect, dose-response, and plausible confounding factors.

^b^ Because the included RCTs had a higher risk of bias, especially with other biases besides blinding of participants, personnel, and outcome assessors.

^c^ Because there was a small number of patients in the analysis with wide CI.

^d^ Because the outcome had significant heterogeneity; however, the degrees of heterogeneity were moderate in all outcomes.

Very low means that our confidence about the result is very little and the actual effect is more probably to be different from our result.

## Discussion

Our systematic review and meta-analysis found statistically significant differences in endpoints of VAS pain and STAI anxiety levels between music and non-music groups and a decrease in VAS scores from baseline that favored the music group. However, we found no significant difference between the two groups in the decrease in STAI levels from the baseline. As far as we know from the literature, this is the first systematic review and meta-analysis that addresses the effect of music on pain and anxiety levels in patients undergoing outpatient hysteroscopy.

Our main finding was supported by the findings of the clinical trial by Law et al. who found that listening to music significantly eliminated the need for analgesics during performing outpatient hysteroscopy [21]. Also, the result of Angioli et al. reinforced the results of this meta-analysis by reporting that pain levels were lower in the music group than non-music group [20]. On the other hand, Mak et al. reported that music showed no positive effect on pain and recommend that a multimodal technique was needed to control pain with or without music which was not consistent with the results of our study [22].

Chan et al. reported that music had a significant effect in lowering the perceived VAS pain by women during colposcopy which further supported the results of our study despite the different gynecological procedures [28]. However, by comparing the results of this meta-analysis to the results of Abdelhakim et al. – a meta-analysis which addressed the effect of music on pain in patients undergoing colposcopy, we found they did not come in agreement with our results. Abdelhakim et al. reported that they found no effect of music therapy in reducing pain levels when compared with the control group [29]. Abdelhakim et al. mentioned that their results could be due to the heterogeneity in the included studies. Also, Abdelhakim et al. reported that there were different cultures in their population and the type of music could not be accepted by different cultures.

Chan et al. – a clinical trial – reported that anxiety state and pain perception were highly correlated [28]. In agreement with our results, they reported that the level of anxiety was significantly lower in the music group than in the non-music group. Also, the results of Yuan et al. came in agreement with our study findings as they reported that music had a significant positive effect on anxiety levels. In addition, they found that other outcomes were not statistically significant like a decrease in the heart rate, less increase in systolic blood pressure, and a smaller pain score change in the music group compared to the non-music group patients which further supports the results of our study [30]. The results of Galal et al. – a systematic review – were similar to ours where they concluded that playing music during colposcopy examination had a significant effect on reducing the levels of pain and anxiety among women undergoing the procedure [31]. Despite the different gynecological procedures in Galal et al. and our study, the idea of lowering pain in response to music was the same in both of the studies. Regarding anxiety, the results of our systematic review were consistent with the results of Angioli et al. where music had a significant effect on reducing the endpoint STAI anxiety score during hysteroscopy. On the other hand, Mak et al. found that music had no positive effect on the endpoint STAI anxiety score which contradicted the results of our study [22]. Also, Abdelhakim et al. found that music had no effect on anxiety during colposcopy which also contradicted the results of this study [29].

The main strength of our study was that to our knowledge this was the first systematic review and meta-analysis that investigated the effect of music in relieving pain and anxiety during the outpatient hysteroscopic procedure.

However, the major limitation of this study is the small number of the included studies – only three – and consequently a relatively low sample size. Another limitation was that the results of all the addressed outcomes were heterogeneous. Also, many confounding factors could affect the results which were not investigated in our study because of the limited included studies or unavailable specific data about these factors in the included studies. These factors can be related to the patients like age, menopausal status, or personal preferences in enjoying specific types of music. The purpose of the procedure also can affect the results whether diagnostic or interventional. Finally, the different types of music used in each study can result in different results. Also, all these factors can explain the heterogeneity of our results which makes us recommend that the following clinical trials should give specific data about these factors and study their association in reducing pain and anxiety outcomes.

According to the GRADE assessment, all the outcomes made very low evidence. The high risk of bias, imprecision, and the presence of significant heterogeneity in the included studies explains these low evidence scores. This limitation could be an indication that the actual results had a high chance to be different from our findings and reduced the ability of this systematic review and meta-analysis to reach a reliable and precise conclusion. However, the observed increased risk of bias in our studies which lowered the evidence of our outcomes come from the inability to blind neither the participants nor the outcome assessors because of the nature of the intervention; however, Mak et al. overcome this by not telling participants about the nature of the intervention which could be applied in the upcoming RCTs [22]. In addition, despite the observed heterogeneities in all outcomes being moderate, this resulted in lowering their evidence. The heterogeneity could be explained by the different cultures and countries in which the included studies were conducted together with the different modes of music that were used in each study. Finally, performing more RCTs can enhance the precision of these results.

Based on that, we recommend that more RCTs addressing this topic should be done to reach a meaningful and reliable conclusion. Also, these RCTs should include a sufficient number of patients.

In conclusion, Music is a potentially promising method for controlling pain for patients undergoing outpatient hysteroscopy.

## Data Availability

The datasets used and/or analyzed during the current study are not publicly available due to the difficulty of the organization of the data to be suitable for publication; however, they are available from the corresponding author upon reasonable request.

## List of Abbreviations

RCTs: Randomized Clinical Trials
VAS: Visual Analogue Scale
STAI: State-Trait Anxiety Inventory
GRADE: score, the Grading of Recommendations Assessment, Development and Evaluation
MD: Mean Difference
CI: Confidence Interval
